# Facile synthesis of nanoparticles-stacked Co_3_O_4_ nanoflakes with catalase-like activity for accelerating wound healing

**DOI:** 10.1093/rb/rbae006

**Published:** 2024-01-25

**Authors:** Yanan Huang, Wanyi Liao, Wenxuan Wang, Tingting Zhang, Yan Zhang, Lei Lu

**Affiliations:** School and Hospital of Stomatology, Wenzhou Medical University, Wenzhou 325027, China; Sichuan Engineering Research Center for Biomimetic Synthesis of Natural Drugs, School of Life Science and Engineering, Southwest Jiaotong University, Chengdu 610031, China; Sichuan Engineering Research Center for Biomimetic Synthesis of Natural Drugs, School of Life Science and Engineering, Southwest Jiaotong University, Chengdu 610031, China; Key Lab of Advanced Technology of Materials of Education Ministry, School of Materials Science and Engineering, Southwest Jiaotong University, Chengdu 610031, China; School and Hospital of Stomatology, Wenzhou Medical University, Wenzhou 325027, China; Sichuan Engineering Research Center for Biomimetic Synthesis of Natural Drugs, School of Life Science and Engineering, Southwest Jiaotong University, Chengdu 610031, China; Sichuan Engineering Research Center for Biomimetic Synthesis of Natural Drugs, School of Life Science and Engineering, Southwest Jiaotong University, Chengdu 610031, China; School of Chemistry, Southwest Jiaotong University, Chengdu, Sichuan 610031, China; School and Hospital of Stomatology, Wenzhou Medical University, Wenzhou 325027, China

**Keywords:** Co_3_O_4_, nanoflake, nanozyme, reactive oxygen species, wound healing

## Abstract

Delayed wound healing caused by excessive reactive oxygen species (ROS) remains a considerable challenge. In recent years, metal oxide nanozymes have gained significant attention in biomedical research. However, a comprehensive investigation of Co_3_O_4_-based nanozymes for enhancing wound healing and tissue regeneration is lacking. This study focuses on developing a facile synthesis method to produce high-stability and cost-effective Co_3_O_4_ nanoflakes (NFs) with promising catalase (CAT)-like activity to regulate the oxidative microenvironment and accelerate wound healing. The closely arranged Co_3_O_4_ nanoparticles (NPs) within the NFs structure result in a significantly larger surface area, thereby amplifying the enzymatic activity compared to commercially available Co_3_O_4_ NPs. Under physiological conditions, it was observed that Co_3_O_4_ NFs efficiently break down hydrogen peroxide (H_2_O_2_) without generating harmful radicals (·OH). Moreover, they exhibit excellent compatibility with various cells involved in wound healing, promoting fibroblast growth and protecting cells from oxidative stress. In a rat model, Co_3_O_4_ NFs facilitate both the hemostatic and proliferative phases of wound healing, consequently accelerating the process. Overall, the promising results of Co_3_O_4_ NFs highlight their potential in promoting wound healing and tissue regeneration.

## Graphical Abstract

**Figure:**
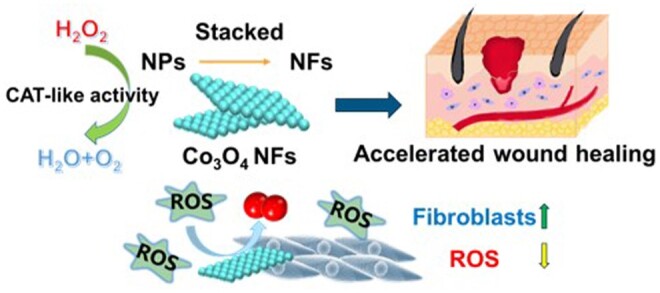

## Introduction

Delayed wound healing accompanied by chronic inflammation is a significant challenge in burn injuries, diabetic foot ulcers and surgical wounds. This issue poses a growing burden on the medical system [1, 2]. Reactive oxygen species (ROS) generated in impaired wounds play a crucial role in cellular homeostasis and physiological regulation. However, excessive ROS accumulation at the wound site leads to oxidative damage, chronic inflammation and hinder tissue repair [3–5]. Therefore, it is essential to regulate the oxidative microenvironment by scavenging excess ROS to accelerate wound healing [6, 7].

Natural antioxidant enzymes such as catalase (CAT), superoxide dismutase (SOD) and glutathione peroxidase are effective in scavenging ROS and modulating oxidative stress [8]. However, their clinical application faces challenges due to inherent characteristics such as low stability, high cost and sensitivity to environmental conditions [9]. On the other hand, nanozymes, which are nanomaterials that mimic enzyme-like functions, offer promising advantages in biomedical applications due to their high stability and cost-effectiveness [10]. Metal oxide-based nanozymes, including Fe_3_O_4_, CeO_2_, CuO, MnO_2_ and Co_3_O_4_, have been developed and extensively studied for their peroxidase-like, oxidase-like, CAT-like and/or SOD-like activities in wound healing and tissue repair [11–16].

Cobalt (Co) is an essential trace element that forms the active center of vitamin B_12_, crucial for multiple biosynthesis and metabolism processes [17, 18]. Co-based materials, such as Co alloys, have been widely used as implantable biomaterials due to their excellent biocompatibility [19, 20]. Recently, Co and its oxide-based nanozymes, particularly Co_3_O_4_ nanoparticles (NPs), have demonstrated CAT-like and peroxidase-like activities in various biomedical applications, including glucose [12, 21] and hydrogen peroxide (H_2_O_2_) detection [22], as well as immunohistochemical assays [23]. Therefore, it is of great interest to explore the beneficial effects of Co-based nanozymes with enzyme-like properties for facilitating wound healing and promoting tissue regeneration [8, 24–26].

In this study, we have synthesized Co_3_O_4_ NPs-stacked Co_3_O_4_ nanoflakes (NFs) with CAT-like activity through a facile one-step synthesis. The closely arranged Co_3_O_4_ NPs in the NFs structure provide a large surface area, enhancing the enzymatic activity compared to commercially available Co_3_O_4_ NPs. Under physiological conditions, Co_3_O_4_ NFs exhibit excellent CAT-like activity and is able to decompose H_2_O_2_ into H_2_O and O_2_ without generation of harmful ·OH. Moreover, Co_3_O_4_ NFs demonstrate excellent cytocompatibility with various cells involved in wound healing, significantly promoting fibroblast growth *in vitro*, and protecting cells from oxidative stress damage in the presence of an oxidative stress environment. Finally, we demonstrate the potential of Co_3_O_4_ NFs to accelerate the wound healing process by facilitating both the hemostatic and proliferative phases using a rat model.

**Figure 1.**
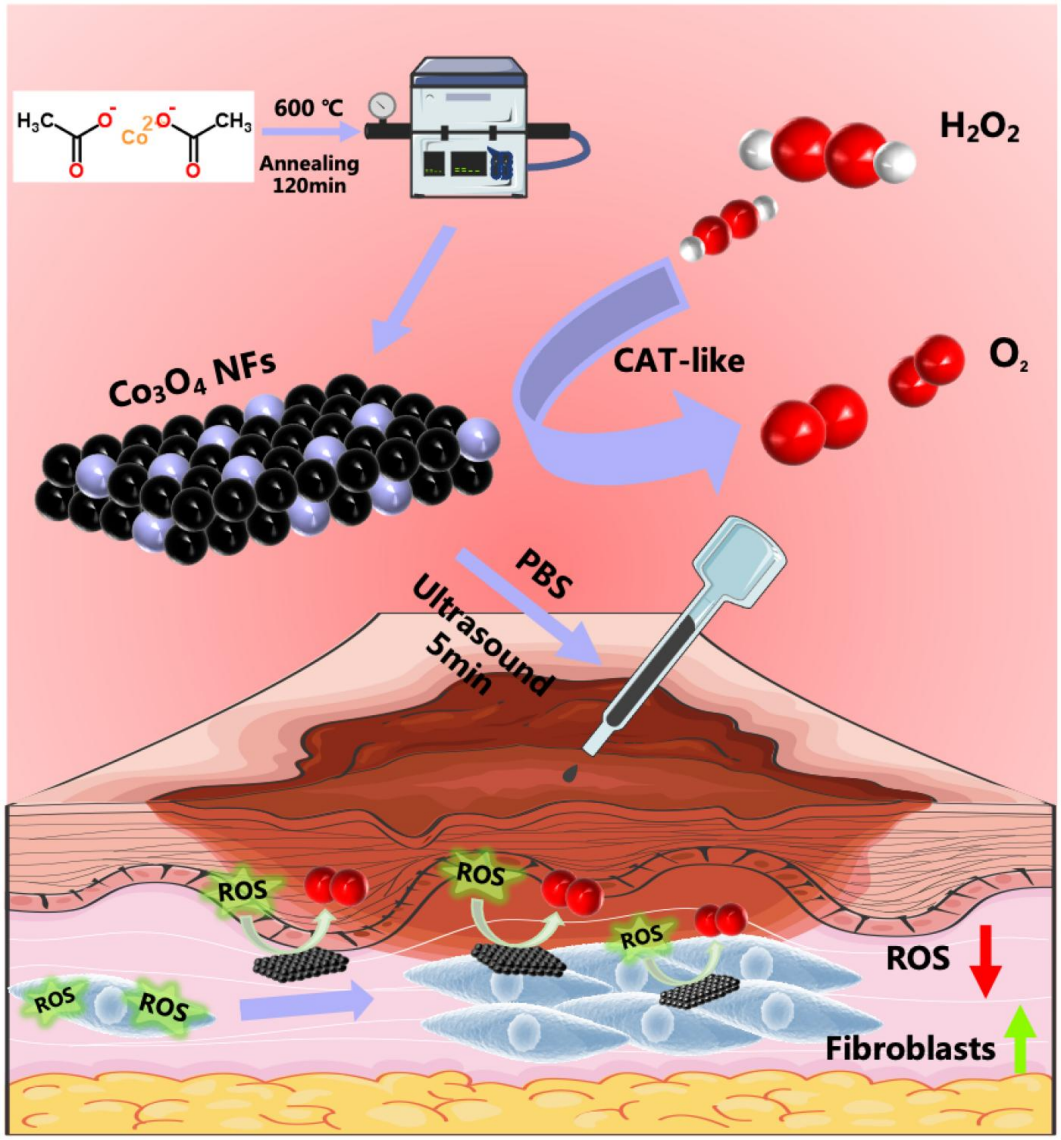
Schematic illustration of the application of Co_3_O_4_ NFs with CAT-like activity and excellent cytocompatibility for promoting wound healing.

In summary, our study focuses on investigating the advantages of the simply synthesized Co_3_O_4_ NFs with CAT-like activity in promoting wound healing and tissue regeneration. The excellent biocompatibility, fibroblast promotion and ability to protect cells from oxidative stress make Co_3_O_4_ NFs a promising candidate for enhancing wound healing processes (Figure 1).

## Experimental section

### Chemicals

Co(CH_3_COO)_2_⋅4H_2_O (99%) was purchased from Adamas-beta Co., Ltd (Shanghai, China). Co_3_O_4_ NPs (99.5%, 100 nm) were purchased from Innochem Technology Co., Ltd (Beijing, China). CAT, CAT activity test kit (ammonium molybdate method), and 3,3′,5,5′-tetramethylbenzidine (TMB) were purchased from Solarbio Science & Technology Co., Ltd (Beijing, China). DMEM-high glucose medium and fetal bovine serum (FBS) were purchased from ScienCell Research Laboratories (San Diego, CA, USA). Cell Counting Kit-8 (CCK-8) was from Dojindo Laboratories (Kumamoto, Japan). Activated partial thromboplastin time (APTT) and prothrombin time (PT) reagents were purchased from Shanghai Sun Biotechnology Co., Ltd (Shanghai, China). Live/Dead Cell Detection Kit and 2,7-dichlorodihydrofluorescein diacetate (DCFH-DA) were purchased from KeyGEN BioTECH Co., Ltd (Jiangsu, China). DAPI and Rhodamine 123 dyes were purchased from Servicebio Technology Co., Ltd (Wuhan, Hubei, China).

### Preparation of Co_3_O_4_ NFs

To prepare Co_3_O_4_ NFs, Co(CH_3_COO)_2_-4H_2_O was placed in a tube furnace and annealed at 600°C for 120 min in air. Then the system was cooled to room temperature, and Co_3_O_4_ NFs were obtained.

### Characterization

The scanning electron microscope (SEM) morphology was observed using FEI Inspect F50. The high-resolution transmission electron microscopy (HRTEM) images were taken using FEI Tecnai G2 Spirit TWIN, X-ray powder diffraction (XRD) pattern was recorded with a Rigaku Ultima IV diffractometer using Cu Kα radiation (λ = 1.5406 Å). The X-ray photoelectron spectroscopy (XPS) was determined using Thermo Scientific Escalab 250xi. The concentration of Co ions was quantified using Inductively coupled plasma-Mass Spectrometry (ICP-MS, PerkinElmer NexION 2000). The hydrodynamic size of NFs was measured by dynamic light scattering (DLS, Malvern, UK).

### Electrochemical measurement

Cyclic voltammetric was performed on a CS150H electrochemical workstation. Then, 5 μl of Co_3_O_4_ NFs colloidal solution (3% PTFE in water) was dropped on the surface of the pre-treated carbon cloth (CC) electrode and dried for 3 h under environmental conditions to obtain the Co_3_O_4_ NF-modified electrode. The electrocatalytic property was investigated in a three-electrode system comprising the Co_3_O_4_ NF-modified electrode as the working electrode, a carbon rod as an auxiliary and a saturated calomel electrode as reference. All experimental solutions were deoxygenated by bubbling highly pure argon for at least 20 min and maintained under argon atmosphere during the measurements.

### CAT-like activity of Co_3_O_4_ NFs

The CAT-like activity of Co_3_O_4_ NFs was assessed using H_2_O_2_ assay kit (Solarbio, Shanghai, China). Different concentrations of Co_3_O_4_ NFs (0, 0.5, 5, 50, 500, 5000 µg/ml) were incubated with 10 mM H_2_O_2_ for 30 min at 37°C, pH 7.4. The concentration of remaining H_2_O_2_ was obtained by calculating the absorbance at 450 nm according to the manufacturer’s instructions. Under the same conditions, the generated oxygen in solutions at different reaction times was measured using a dissolved oxygen meter (JPSJ-605F, LEICI Auto Industry Co., Ltd, Shanghai, China). The kinetic assays were performed by testing 50 µg/ml of Co_3_O_4_ NFs with different concentrations (0.1, 1, 10, 100 mM) of H_2_O_2_. Michaelis–Menten constants were calculated from the Michaelis–Menten saturation curves of GraphPad Prism 8.0 (GraphPad Software).

### pH-switchable peroxidase-like activity of Co_3_O_4_ NFs

The peroxidase-like activity of the Co_3_O_4_ NFs was measured by TMB colorimetry. Different concentrations of Co_3_O_4_ NFs (0, 0.5, 5, 50, 500, 5000 µg/ml) were incubated with 10 mM H_2_O_2_ for 30 min at 37°C, pH 4.0 and pH 7.4, respectively. The supernatant was collected by centrifuging at 6000 rpm for 5 min. The colorless TMB can be converted into blue ox-TMB by ·OH. The generated ·OH concentration in the supernatant was obtained by calculating the absorbance at 650 nm according to the manufacturer’s instructions.

### Evaluation of long-term and sterilization stability of Co_3_O_4_ NFs

A 30-ml portion of 50 μg/ml Co_3_O_4_ NFs aqueous suspension was prepared in PBS at pH 7.4 and the solution was slowly stirred at 37°C. At time points of 0, 3 and 14 days, respectively, the suspension was centrifuged. Then, 5 ml of supernatant was collected, and 5 ml of fresh PBS was added to maintain a consistent volume. The concentration of Co ions was determined by ICP–MS and filtered with a 0.2-μm filter prior to testing.

The Co_3_O_4_ NFs and 2000 U CAT were autoclaved, sterilized in 75% alcohol and allowed to stand for 14 days (37°C, pH 7.4). The residual enzyme activity of the treated Co_3_O_4_ NFs and 2000 U CAT was assayed with a H_2_O_2_ assay kit.

### Cell culture

Fibroblast cells (L929) were cultured in DMEM with 10% FBS and 1% penicillin/streptomycin. Endothelial cells (ECs) were cultured in F12 with 10% FBS and 1% penicillin/streptomycin, and smooth muscle cells (SMCs) were cultured in SMCM: F12 (1:1) with 10% FBS and 1% penicillin/streptomycin. Cells are grown to 80–90% at 37°C and 5% CO_2_ incubator for passaging with trypsin digestion.

### Cell viability assessment

L929, ECs and SMCs were first seeded in 96-well plates at 5000 cells/well, respectively. After 24 h, the medium was replaced with fresh medium containing different concentrations of sterilized Co_3_O_4_ NFs (0, 0.5, 5, 50, 500, 5000 µg/ml). After 24 and 72 h of incubation, the cell viability was examined by using a CCK-8 kit.

The cell morphology was observed by fluorescence microscopy (CKX53, OLYMPUS, Japan), after three times washing with saline, fixation by 2.5% glutaraldehyde and being stained with DAPI and Rhodamine 123.

### Cellular ROS-scavenging assessment of Co_3_O_4_ NFs

After 24 h of adhesion of L929 in 96-well plates, fresh medium containing 800 µM H_2_O_2_ and 50 µg/ml Co_3_O_4_ NFs was replaced, 2000 U CAT was used as positive control. After 6 h of co-treatment, the plates were washed two to three times and cell death was immediately observed under a fluorescent microscope using the Live/Dead Cell Detection Kit (KeyGE, Jiangsu, China). The intracellular ROS content was detected using the fluorescent probe DCFH-DA (Beyotime, Shanghai, China).

### Hemolysis assay

The suspension of red blood cells (RBCs) was obtained from fresh (Sprague-Dawley) SD rat blood through centrifugation at 3000 rpm for 15 min. The RBCs were then gently washed and diluted using a saline solution. Subsequently, 20 μl of the RBC solution was mixed with 1 ml of various concentrations of Co_3_O_4_ NFs (0.5, 5, 50, 500, 5000 µg/ml) in PBS. Simultaneously, 20 μl of the RBCs solution was mixed with 1 ml of PBS and deionized water to serve as the negative control and the positive control, respectively. After incubating at 37°C for 3 h, the supernatant from all groups was collected *via* centrifugation at 3000 rpm for 15 min. The absorbance at 540 nm was measured for each sample using a microplate reader. The hemolysis rate was then calculated using the following equation:

$$\text{Hemolysis ratio}\;\left(\%\right)=\left({\text{OD}}_{\text{test}}-{\text{OD}}_{\text{neg}}\right)/\left({\text{OD}}_{\text{pos}}-{\text{OD}}_{\text{neg}}\right)\;*\;100\%$$

where OD_test_ is the OD value of the Co_3_O_4_ NFs group, OD_neg_ is the OD value of the negative control group and OD_pos_ is the OD value of the positive control group.

### Clotting time assays

Fresh rabbit blood was collected with anticoagulant sodium citrate and centrifuged at 2000 r/min for 10 min to obtain platelet-poor plasma. The coagulation time of Co_3_O_4_ NFs was evaluated using PT and APTT kits.

### Wound healing *in vivo*

All animal experiments were approved by the Animal Research Ethics Committee of Wenzhou Medical University (ethical approval No. xmsq2022-0845). Male SD rats (200 g) were acclimatized to room temperature and normal humidity for 1 week before the experiment. General anesthesia was executed for all rats before the experiment, and dorsal skin was shaved and then sterilized with 70% ethanol. Four circular full-thickness skin wounds of approximately 1 cm in diameter were excised from the back of the rats, then PBS with different concentrations (0, 5, 50 µg/ml) of Co_3_O_4_ NFs were applied over the wounds. Elizabethan Collar was applied to each rat, and the wound healing progress was observed on Days 0, 7 and 14.

### Histological analysis

Rats were executed on Days 7 and 14, respectively. Tissue samples were collected and fixed with 4% w/v paraformaldehyde. Tissue sections were then prepared by paraffin embedding and stained for collagen fibers (Masson), hematoxylin and eosin (H&E). To evaluate the organ toxicity of Co_3_O_4_ NFs, heart, liver, spleen, lung and kidney of rats at Day 14 were also collected for H&E staining. The stained sections were observed and images were captured using light microscopy (CKX53, OLYMPUS, Japan).

### Statistical analysis

All results are presented as mean ± standard deviation in this study. Statistical significance was assessed by adopting a one-way analysis of variance via SPSS software. All the tests in this study were executed at least three times with more than four duplicate samples.

## Results and discussion

### Characterization of Co_3_O_4_ NFs

**Figure 2.**
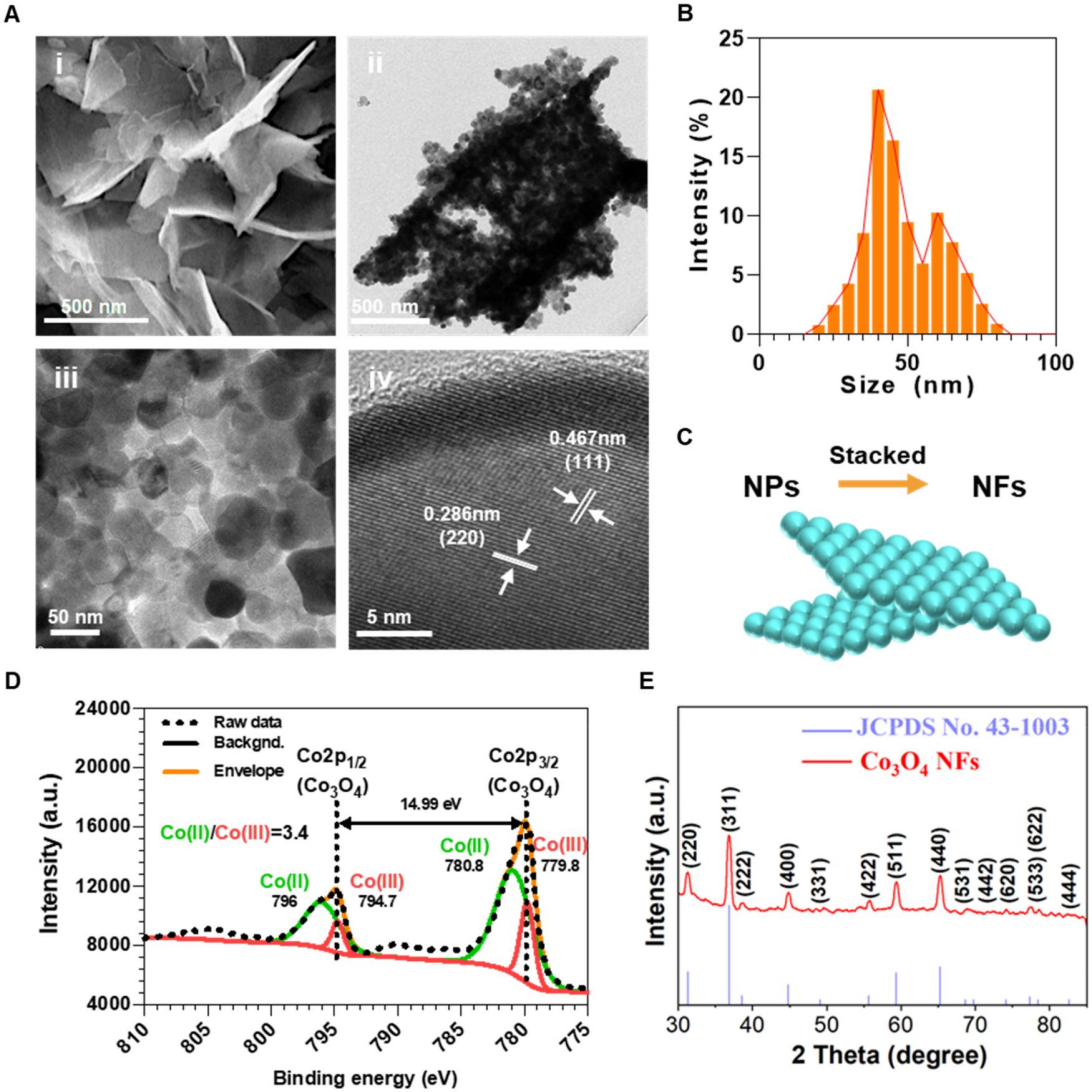
Characterization of Co_3_O_4_ NFs. SEM (**A-i**) and HRTEM (**A-ii** to **A-iv**) images of Co_3_O_4_ NFs; (**B**) size statistics; (**C**) schematic of the stacked Co_3_O_4_ NPs to Co_3_O_4_ NFs; XPS analysis (**D**) and XRD spectrum (**E**) of Co_3_O_4_ NFs.

Co_3_O_4_ NFs were fabricated by a simple one-step method. As the SEM image is shown in Figure 2A-i, the morphologies of Co_3_O_4_ NFs are nanosheets with a thickness of about 35 nm. TEM images of Co_3_O_4_ NFs revealed that the NFs were composed of NPs with a diameter of around 42 ± 6.5 nm (Figure 2A-ii and A-iii, and the size distribution of NPs is shown in Figure 2B). The HRTEM images showed that the lattice fringes with spacing of 0.467, 0.286 nm correspond to the (111), (220) planes of Co_3_O_4_ (Figure 2A-iii). The hydrodynamic size of Co_3_O_4_ NFs in PBS was about ∼1001 nm (Pdi: 0.33, Supplementary Figure S1). Thus, we speculate that the Co_3_O_4_ NFs are stacked by a single layer of Co_3_O_4_ NPs. The XPS spectrum of Co 2p (Figure 2D) shows that the difference in binding energy between the peaks of Co 2p3/2 and Co 2p1/2 is 14.99 eV. Among them, the peaks at 780.8 eV and 796 eV correspond to Co^2+^, and the peaks at 779.8 eV and 794.7 eV correspond to Co^3+^, confirming the successful synthesis of Co_3_O_4_ NFs [22]. XRD result also confirmed the product as a pure cubic spinal structure of Co_3_O_4_ NFs (JCPDS no. 43-1003, Figure 2E) [27, 28], which was consistent with the HRTEM result (Figure 2A-iii).

To assess the electrocatalytic activity of Co_3_O_4_ NFs as CAT, its ability to catalyze the reduction of H_2_O_2_ was examined. The cyclic voltammogram of the Co_3_O_4_ NF-modified CC electrode is shown in Supplementary Figure S2. Notably, a reduction peak at −0.25 V is observed, indicating that Co_3_O_4_ NFs possess the capability to catalyze the reduction of H_2_O_2_.

### CAT-like activity of Co_3_O_4_ NFs

**Figure 3.**
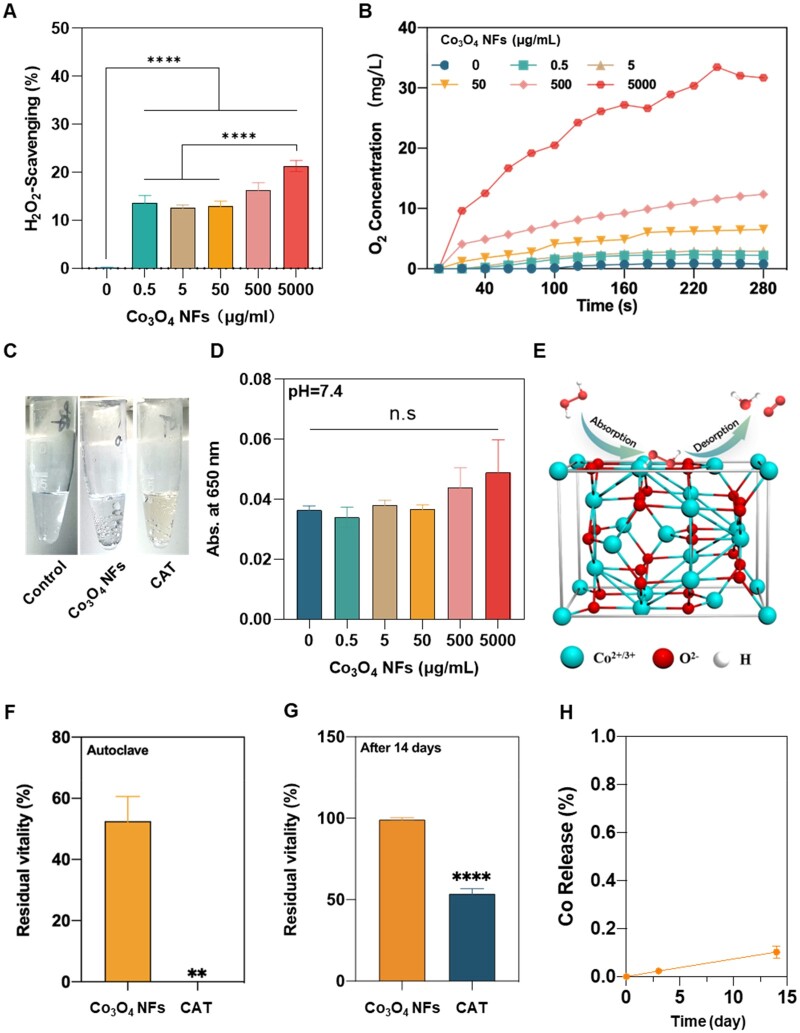
CAT-like ROS-scavenging activities and stability of Co_3_O_4_ NFs. (**A**) Clearance of 10 mM H_2_O_2_ by Co_3_O_4_ NFs; (**B**) the generated dissolved oxygen after Co_3_O_4_ NFs reacted with 100 mM H_2_O_2_; (**C**) the observed O_2_ bubble generation; (**D**) determination of the H_2_O_2_ decomposition products ·OH at pH 7.4; (**E**) schematic diagram of the catalytic mechanism; H_2_O_2_ scavenging activity of Co_3_O_4_ NFs and CAT after autoclaving (**F**) and 14 days of incubation (**G**); (**H**) Co ion release during 14-d incubation. **p < 0.01, ****p < 0.0001, n.s: no significance.

The scavenging activity of Co_3_O_4_ NFs for H_2_O_2_ was found to be concentration-dependent (Figure 3A). The dissolved O_2_ was also measured, which also showed a Co_3_O_4_ NFs concentration-dependent O_2_ generation (Figure 3B). A large number of bubbles were generated during the reaction, which correspond to the previous results (Figure 3C). TMB colorimetry was used to determine the presence of ·OH in the supernatant of the reaction solutions. As shown in Figure 3D, there is no ox-TMB produced in all groups at pH 7.4. That is, the products of H_2_O_2_ decomposition by Co_3_O_4_ NFs are H_2_O and O_2_ without the generation of ·OH under physiological conditions. Therefore, Co_3_O_4_ NFs possess CAT-like activity under physiological conditions. Moreover, H_2_O_2_ decomposition by Co_3_O_4_ NFs in an acidic environment (pH 4) was also determined, which showed a significant concentration-dependent ·OH generation (Supplementary Figure S3), indicating the pH-switchable peroxidase-like activity of Co_3_O_4_ NFs [21].

The CAT-like activity of Co_3_O_4_ NFs was further investigated using steady-state kinetics, and Michaelis–Menten curves were used to obtain kinetic data by varying the concentration of one substrate while keeping the concentration of the other substrate constant. By fitting the data to the Michaelis–Menten equation to determine the catalytic parameters (Supplementary Table S1):

$$\frac{1}{\nu \;}=\left(\frac{K_{m}}{V_{\text{max}}}\right)\cdot \left(\frac{1}{\left[S\right]}\right)+\frac{1}{\;V_{\text{max}}}$$

where *V* is the initial velocity, [*S*] is the substrate concentration, *K_m_* is the Michaelis–Menten constant and *V*_max_ is the maximum reaction velocity.

It was found that the decomposition of H_2_O_2_ induced by Co_3_O_4_ NFs followed Michaelis–Menten kinetics (Supplementary Figure S4). Where *K_m_* represents the affinity of the enzyme for the substrate and a smaller *K_m_* value indicates a stronger affinity between the enzyme and the substrate. This means that Co_3_O_4_ NFs have a higher affinity for H_2_O_2_ than natural peroxidases and require a lower concentration of H_2_O_2_ to obtain the maximum reaction rate. The higher catalytic constant *k*_cat_ value indicates higher enzyme activity [22, 23], thus the catalytic activity of Co_3_O_4_ NFs is slightly lower than that of natural CAT (Supplementary Table S1). The possible catalytic mechanism is illustrated in Figure 3E.

Sterilization stability is crucial for ensuring the safety and effectiveness of wound dressings, particularly for clinical use and cost efficiency. Like other natural enzymes, natural CAT has poor stability, which limits its application. By comparing the long-term stability and sterilization stability of CAT with Co_3_O_4_ NFs, it was found that Co_3_O_4_ NFs can withstand sterilization methods commonly used in medicine, such as autoclaving. In contrast, the activity of CAT was greatly reduced after sterilization (Figure 3E). In addition, after 14-day incubation of Co_3_O_4_ NFs and CAT under physiological conditions, the activity of Co_3_O_4_ NFs was found to be unaffected (Figure 3F); however, the activity of CAT was reduced to ∼50% of the original. Meanwhile, there were almost no Co ions released during 14-day incubation (Figure 3G).

Additionally, the comparison of catalytic activities between Co_3_O_4_ NFs and commercially available Co_3_O_4_ NPs is presented in Supplementary Figure S5. Similarly, the commercial Co_3_O_4_ NPs exhibited concentration-dependent generation of O_2_ (Supplementary Figure S5A). However, it was observed that the catalytic activities of the Co_3_O_4_ NPs significantly decreased at lower concentrations (0.5–50 μg/ml), whereas the Co_3_O_4_ NFs remained steady (Supplementary Figure S5B). This difference can be attributed to the enhanced surface area and greater number of catalytic sites present in the Co_3_O_4_ NFs.

### *In vitro* cell compatibility and ROS-scavenging activity of Co_3_O_4_ NFs

**Figure 4.**
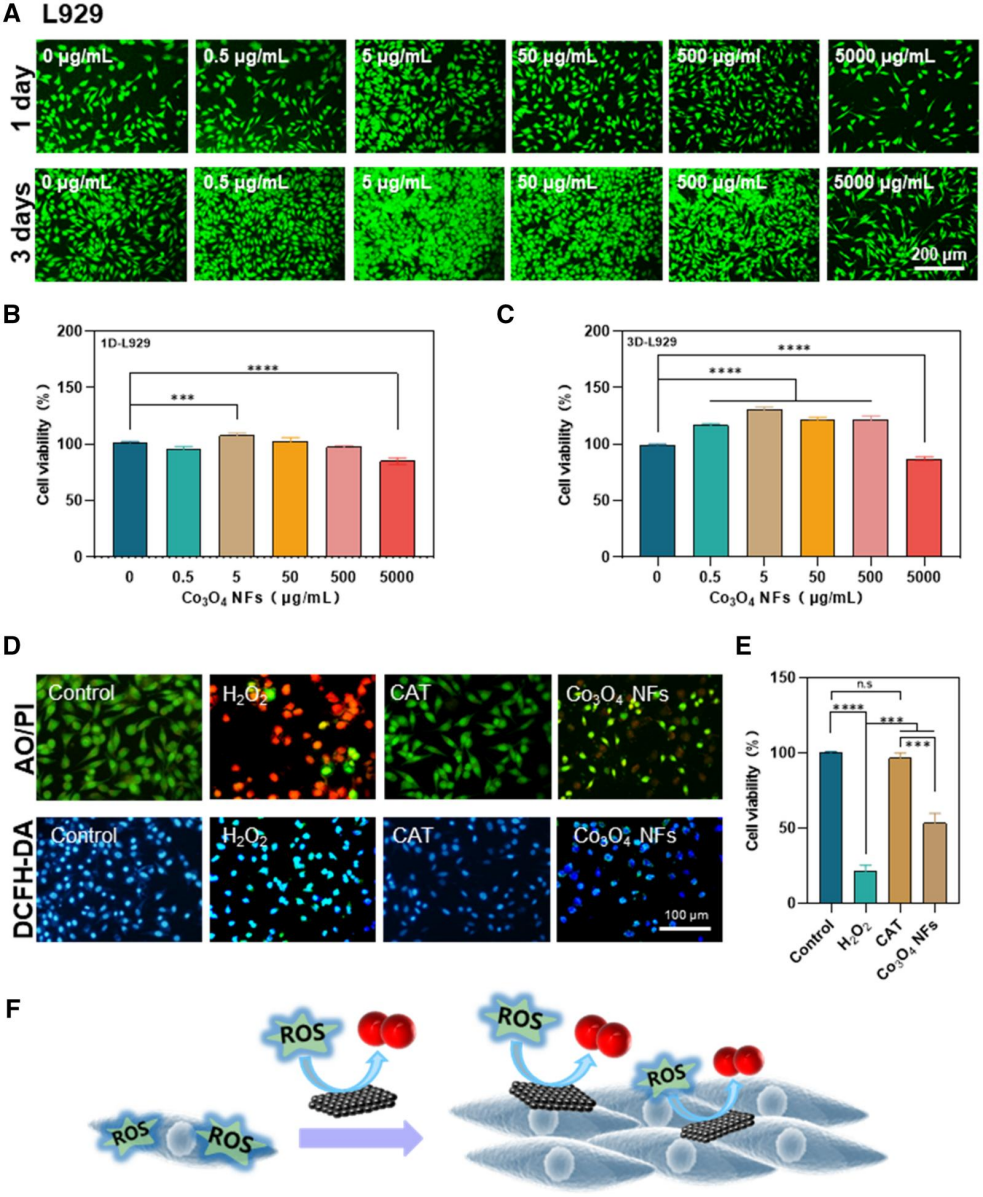
Co_3_O_4_ NFs Promote L929 cell proliferation and reduce cellular oxidative stress. (**A**) Florescent microscopy of L929 cells co-cultured with different concentrations (0, 0.5, 5, 50, 500 and 5000 µg/ml) of Co_3_O_4_ NFs for 1 and 3 days; (**B, C**) cell viability of the co-cultured L929 cells on Day 1 or Day 3, respectively; (**D**) expression of intracellular ROS (DCFH-DA) and live-dead state (AO/EB) of L929 cells co-cultured with 50 µg/ml Co_3_O_4_ NFs and 800 µM H_2_O_2_ for 6 h (live cells were represented by green, dead cells by red, nuclei in blue and intracellular ROS in blue-green); (**E**) statistical analysis of the number of live cells after incubation; (**F**) schematic illustration of the ROS-scavenging mechanism of Co_3_O_4_ NFs. ***p < 0.001, ****p < 0.0001.

The *in vitro* cell compatibility of Co_3_O_4_ NFs was assessed using L929, ECs and SMCs, all of which play crucial roles in the wound healing process. The cell activity of L929 cells was assessed after co-culturing them with different concentrations (0, 0.5, 5, 50, 500, 5000 µg/ml) of Co_3_O_4_ NFs for 1 and 3 days (Figure 4A–C). The statistical analysis revealed that Co_3_O_4_ NFs exhibited a promotion in the proliferation of L929 cells when administered at concentrations ranging from 0.5 to 500 µg/ml. However, the group treated with 5000 µg/ml of Co_3_O_4_ NFs showed slight cytotoxicity, which was consistent with the observations of cell morphologies using fluorescence microscopy.

The *in vitro* antioxidant performance of Co_3_O_4_ NFs, specifically their cellular ROS-scavenging activity and protective effects under oxidative stress conditions, was further investigated. As depicted in Figure 4D, treatment of L929 cells with H_2_O_2_ alone resulted in a significant increase in intracellular ROS levels, leading to reduced cell survival. However, Co_3_O_4_ NFs demonstrated a remarkable cytoprotective effect against H_2_O_2_-induced oxidative stress, evidenced by a reduction in intracellular ROS levels, ultimately promoting enhanced cell viability (Figure 4E). These results strongly indicate that Co_3_O_4_ NFs possess the ability to effectively scavenge ROS within a short period of time, consequently shielding cells from oxidative damage (Figure 4F).

**Figure 5.**
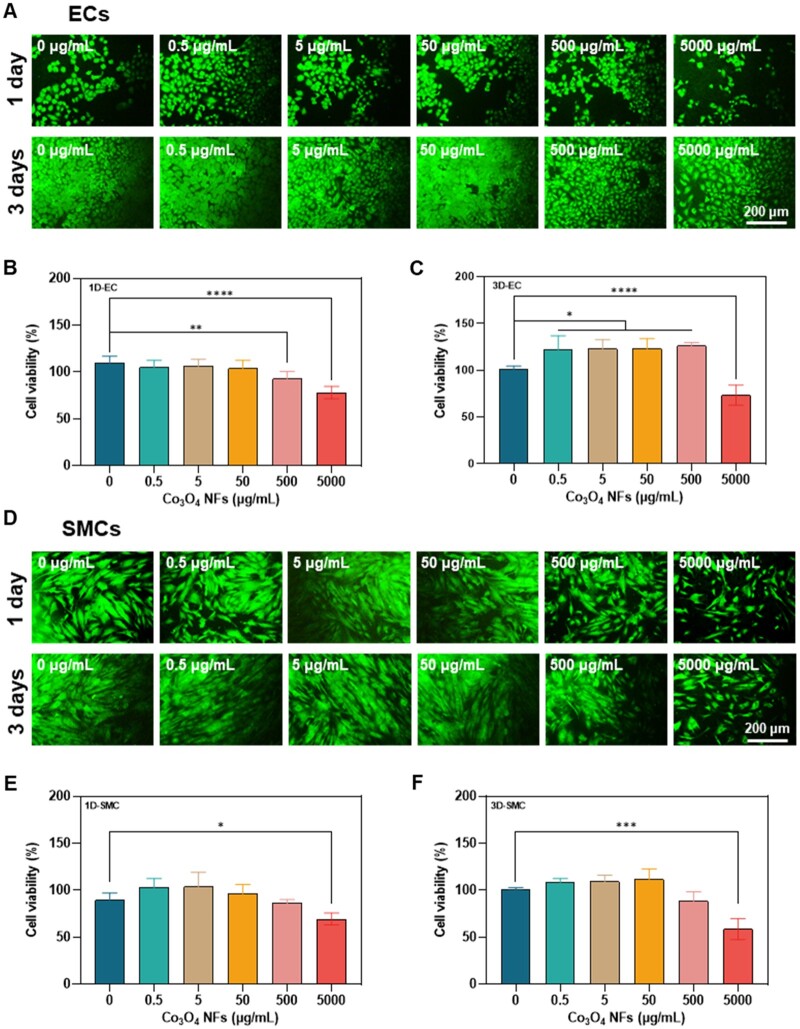
Cytocompatibility assessment of Co_3_O_4_ NFs by ECs and SMCs. Florescent microscopy of ECs (**A**) and SMCs (**D**) co-cultured with different concentrations (0, 0.5, 5, 50, 500 and 5000 µg/ml) of Co_3_O_4_ NFs for 1 and 3 days; cell viability of the co-cultured ECs (**B**, **C**) and SMCs (**E**, **F**) on Day 1 or Day 3, respectively. *p < 0.05, **p < 0.01, ***p < 0.001, ****p < 0.0001.

Besides, the results indicated that Co_3_O_4_ NFs demonstrated good cytocompatibility with ECs and SMCs as well, both of which play a crucial role in vascularization [29, 30] (Figure 5). Moreover, the *in vitro* biosafety of Co_3_O_4_ NFs with RBCs was assessed through a hemolytic experiment. The hemolysis rates of Co_3_O_4_ NFs at various concentrations were found to be below 3% (Supplementary Figure S6).

### Co_3_O_4_ NFs accelerated coagulation *in vitro*

To investigate the impact of Co_3_O_4_ NFs on the coagulation process, experiments utilizing PT and APTT clotting time kits were performed. The results demonstrated a notable reduction in the clotting time of fresh plasma when Co_3_O_4_ NFs were introduced (Supplementary Figure S7). It was speculated that the accelerated blood coagulation was facilitated by enhanced adhesion and activation of fibrinogen on the surface of Co_3_O_4_ NFs.

### Co_3_O_4_ NFs promoted wound healing *in vivo*

**Figure 6.**
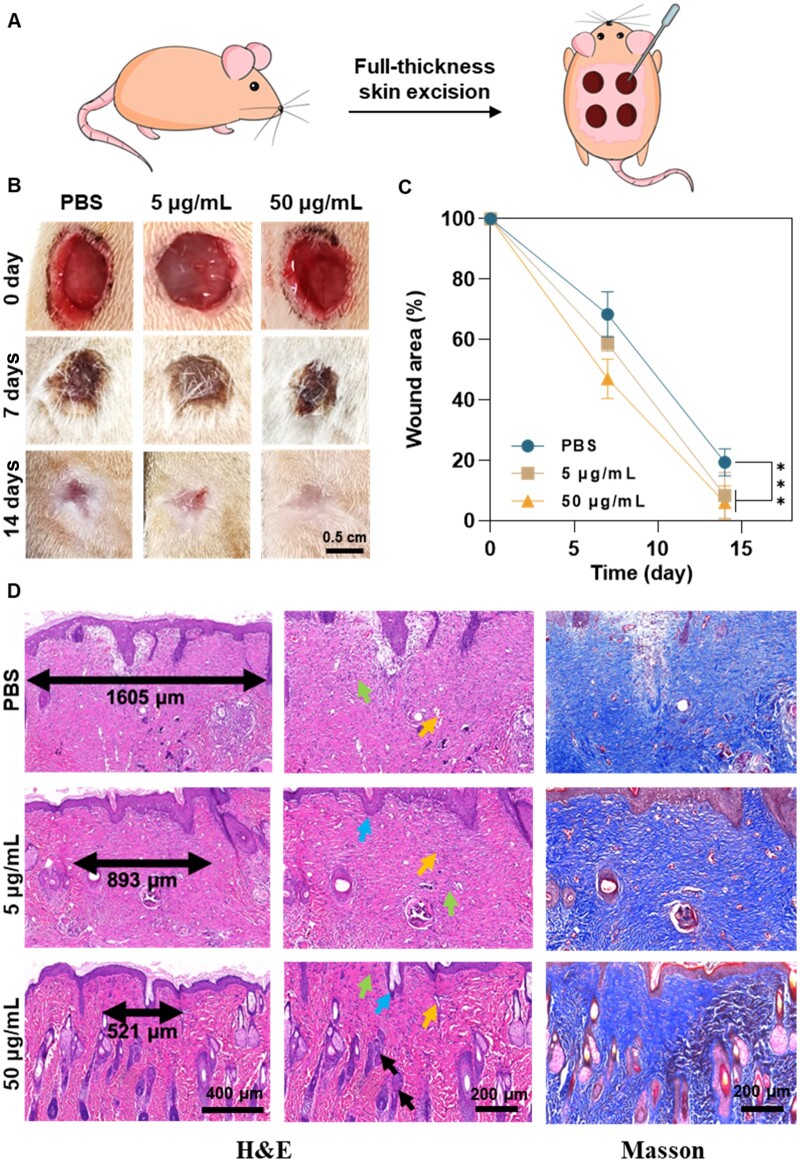
*In vitro* evaluation of Co_3_O_4_ NFs for wound healing. (**A**) Schematic representation of animal experiments. (**B**) Representative images of wound repair on the Day 7 and 14 for the PBS group, 5 µg/ml Co_3_O_4_ NFs group and 50 µg/ml Co_3_O_4_ NFs group. (**C**) Statistical analysis depicting the wound closure. (**D**) Histological staining (H&E, Masson) of the wound on Day 14: green arrow: fibroblasts, blue arrow: epithelial tissue, yellow arrows: capillaries, black arrows: hair follicles.

The effectiveness of Co_3_O_4_ NFs in promoting wound healing was assessed *in vivo* utilizing a full-thickness skin excision SD rat model (Figure 6A). Different concentrations of Co_3_O_4_ NFs suspensions were applied to the skin wounds, with PBS serving as the control group. In Figure 6B and C, it can be observed that Co_3_O_4_ NFs considerably enhanced the rate of wound healing compared to the PBS treatment. Both 5 and 50 μg/ml of Co_3_O_4_ NFs resulted in almost complete closure by Day 14 after treatment (91.7 ± 7.6% and 94.0 ± 5.6%, respectively), whereas the PBS group only achieved 80.7 ± 4.5% closure, indicating a significantly slower healing process.

To evaluate the tissue formation of the healed wounds on Day 14, H&E and Masson staining analyses were performed (Figure 6D). The black double-headed arrow represents the wound edge between the newly formed tissue and the original tissue (Figure 6D). The PBS group has two to three times larger wound edge compared to groups treated with Co_3_O_4_ NFs, which is consistent with the previous observation (Figure 6B and C). Specially, all groups exhibited the basic structures of the epidermis and dermis, along with ample capillaries (yellow arrows) and fibroblasts (green arrows) (Figure 6D). Notably, the PBS group displayed less collagen tissue (blue, indicated by Masson staining, Figure 6D) and lacked mature epithelial structures (blue arrows), whereas the Co_3_O_4_ NFs treated groups displayed abundant collagen tissue. Additionally, papillary epithelial structures (blue arrows) were observed in the Co_3_O_4_ NF-treated groups, indicating the completion of the re-epithelialization stage and the resemblance of the wound tissue to normal tissue. However, hair follicles (black arrows) were found mainly in the group treated with 50 µg/ml Co_3_O_4_ NFs. Furthermore, examination of the organs of both normal and wound model mice revealed that Co_3_O_4_ NFs were not toxic or accumulated in the organ (Supplementary Figure S8). These findings demonstrate that 50 µg/ml Co_3_O_4_ NFs are biocompatible and facilitate the coagulation, proliferation and re-epithelialization phases of wound healing.

## Conclusion

The Co_3_O_4_ NFs with enhanced CAT-like catalytic activity were synthesized through a simple one-step process. Under physiological conditions, these nanozymes effectively decompose H_2_O_2_ into water and O_2_ without generating harmful ·OH. *In vitro* results indicated that Co_3_O_4_ NFs exhibit excellent cytocompatibility, promoting fibroblast growth and protecting cells from oxidative stress. Moreover, they are shown to accelerate wound healing by facilitating both the hemostatic and proliferative phases in a rat model. In addition, we noticed that they also process a pH-switchable peroxidase-like activity, which may benefit future exploration in anti-cancer or anti-microbial applications in acidic environment. In addition, future studies should also aim to investigate the following aspects: (i) Enhancing the CAT-like and peroxidase-like activity of Co_3_O_4_ NFs through materials design strategies, such as introducing heterostructures. (ii) Systematically evaluating the potential applications of Co_3_O_4_ NFs in the biomedical field by comparing them with other metal oxides, such as Mn- and Fe-based nanozymes. (iii) Exploring the SOD-like activity of Co_3_O_4_ NFs and assessing their potential applications in various fields. Overall, this research highlights the potential of Co_3_O_4_ NFs as catalytic nanozymes in the field of wound healing and tissue regeneration, offering exciting possibilities to enhance the healing process and improve patient outcomes.

## Supplementary Material

rbae006_Supplementary_Data
