# Supplementary material for: Facile synthesis of nanoparticles-stacked Co3O4 nanoflakes with catalase-like activity for accelerating wound healing
Source: Regen Biomater. 2024 Jan 25;11:rbae006. doi: 10.1093/rb/rbae006 (PMC10902680; doi:10.1093/rb/rbae006)
Supplement: rbae006_Supplementary_Data [file rbae006_supplementary_data.docx]

Supplementary Materials

**Facile Synthesis of Nanoparticles-Stacked Co_3_O_4_ Nanoflakes with Catalase‐like Activity for Accelerating Wound Healing**

Yanan Huang^1, 2^, Wanyi Liao^2^, Wenxuan Wang^3^, Tingting Zhang^1, 2^, Yan Zhang^2, 4*^, Lei Lu^1*^

*1. School and Hospital of Stomatology, Wenzhou Medical University, Wenzhou, 325027, China.*

*2. Sichuan Engineering Research Center for Biomimetic Synthesis of Natural Drugs, School of Life Science and Engineering, Southwest Jiaotong University, Chengdu 610031, China.*

*3. Key Lab of Advanced Technology of Materials of Education Ministry, School of Materials Science and Engineering, Southwest Jiaotong University, Chengdu, 610031, China.*

*4. School of Chemistry, Southwest Jiaotong University, Chengdu, Sichuan 610031, China*

*** Corresponding authors:**

Lei Lu

School and Hospital of Stomatology, Wenzhou Medical University, Wenzhou, 325027, China.

Email address: [llu2@foxmail.com](mailto:llu2@foxmail.com)

Yan Zhang

Sichuan Engineering Research Center for Biomimetic Synthesis of Natural Drugs, School of Life Science and Engineering, Southwest Jiaotong University, Chengdu 610031, China; School of Chemistry, Southwest Jiaotong University, Chengdu, Sichuan 610031, China

Email address: zyzw@swjtu.edu.cn

| **Table S1.** Kinetic parameters of Co_3_O_4_ NFs. | | | | |
| --- | --- | --- | --- | --- |
| **Samples** | **E (mM)** | **K_m_ (mM)** | **V_max_ (mM min^-1^)** | **K_cat_ (min^-1^)** |
| CAT | 0.90 | 59.78 | 1.92 | 2.13 |
| Co_3_O_4_ NFs | 0.25 | 3.03 | 0.03 | 0.12 |

[E] is the initial molar concentration of the reactant substance; K_cat_ is the catalytic constant; K_cat_ = V_max_/[E].

**Figure S1. The size distribution of Co_3_O_4_ NFs in PBS.**


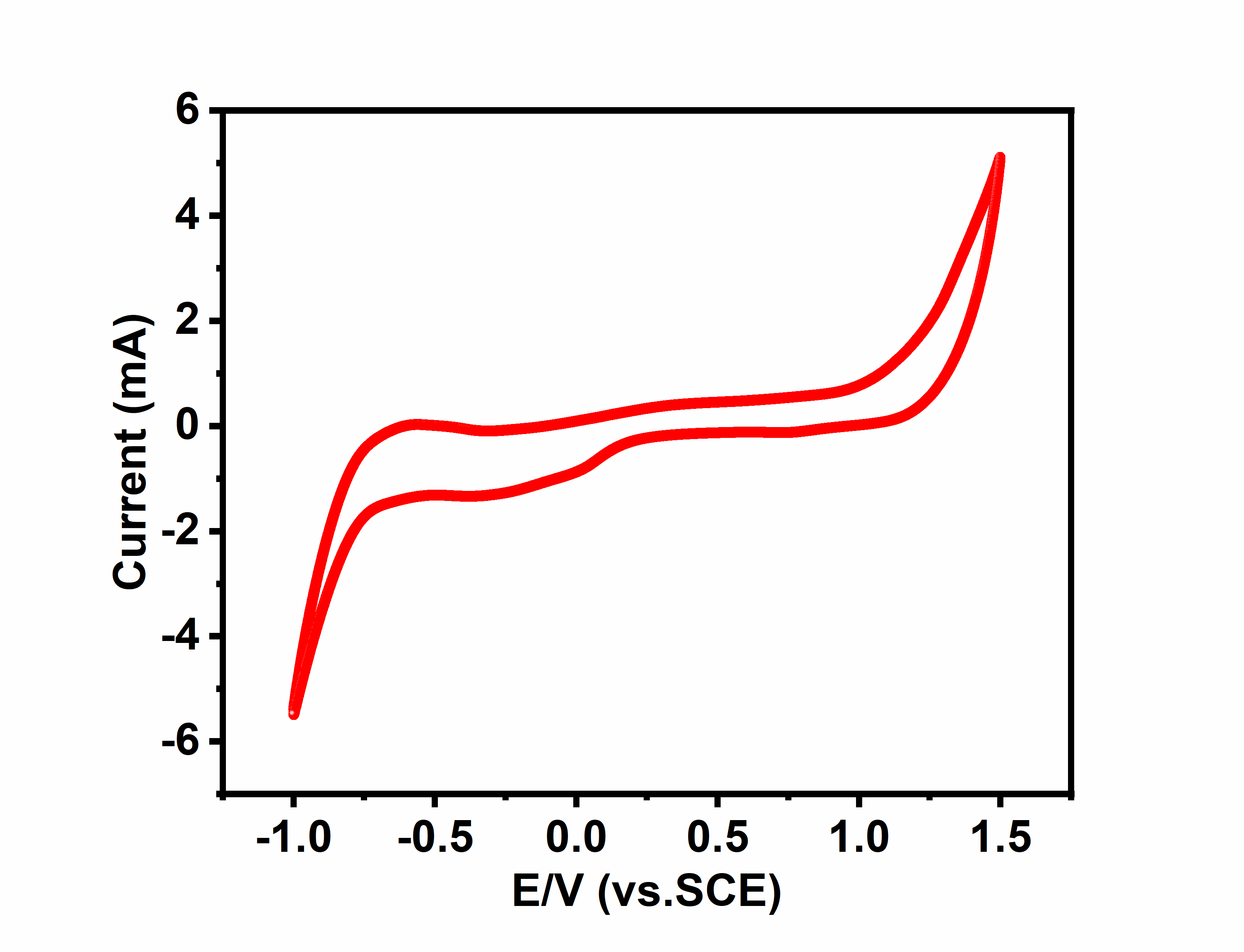


**Figure S2. The cyclic voltammogram of the Co_3_O_4_ NFs modified carbon cloth electrode.**

**Figure S3. Determination of the H_2_O_2_ decomposition products ·OH at pH 4.**


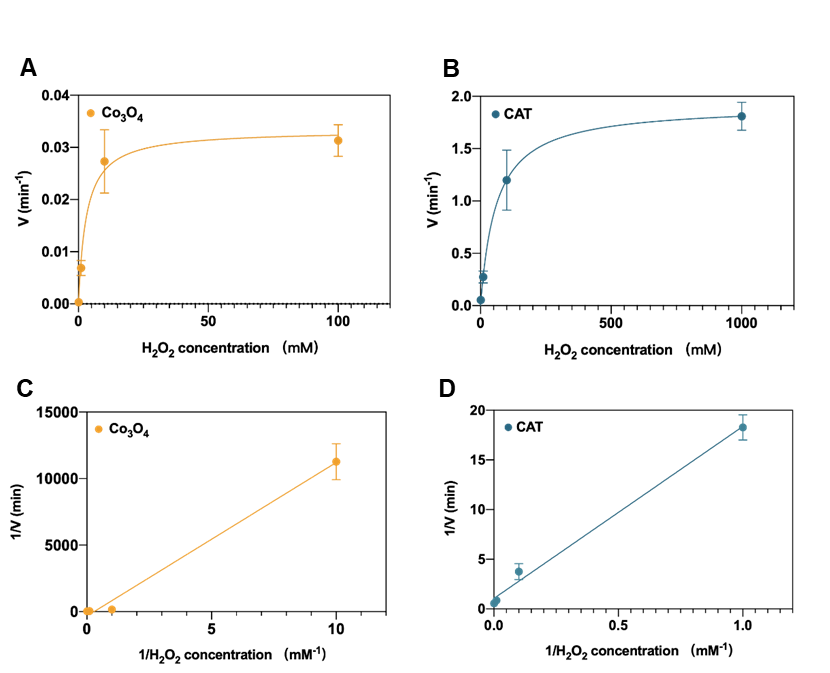


**Figure S4. Michaelis-Menten kinetics of Co_3_O_4_ NFs.** The Michaelies-Menten (A, B) and Lineweaver-Burk (C, D) of Co_3_O_4_ NFs and CAT.


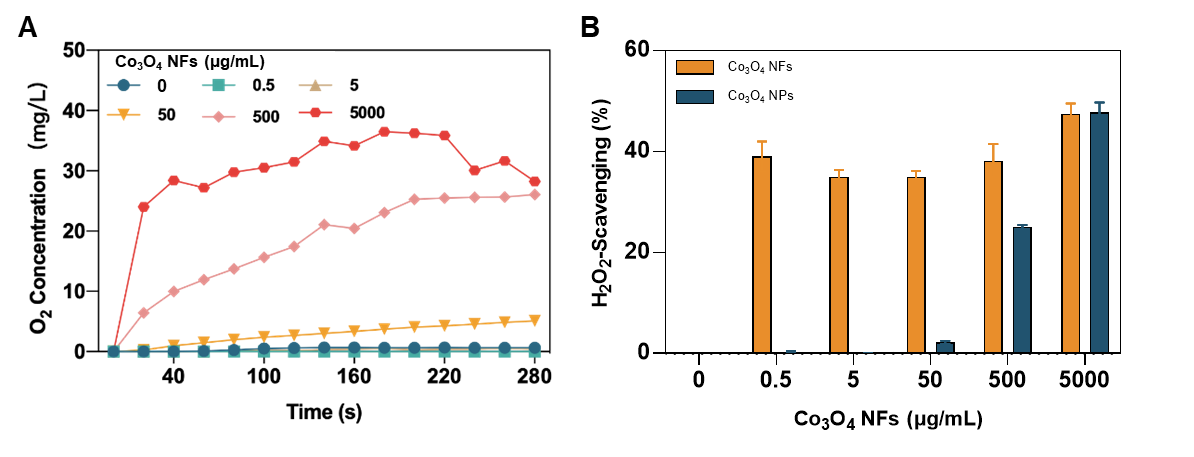


**Figure S5. The comparation of the CAT‐like ROS scavenging activities of Co_3_O_4_ NFs and Co_3_O_4_ NPs.** (A) The generated dissolved oxygen after Co_3_O_4_ NFs and Co_3_O_4_ NPs reacted with 100 mM H_2_O_2_; (B) clearance of 10 mM H_2_O_2_ by Co_3_O_4_ NFs and Co_3_O_4_ NPs.


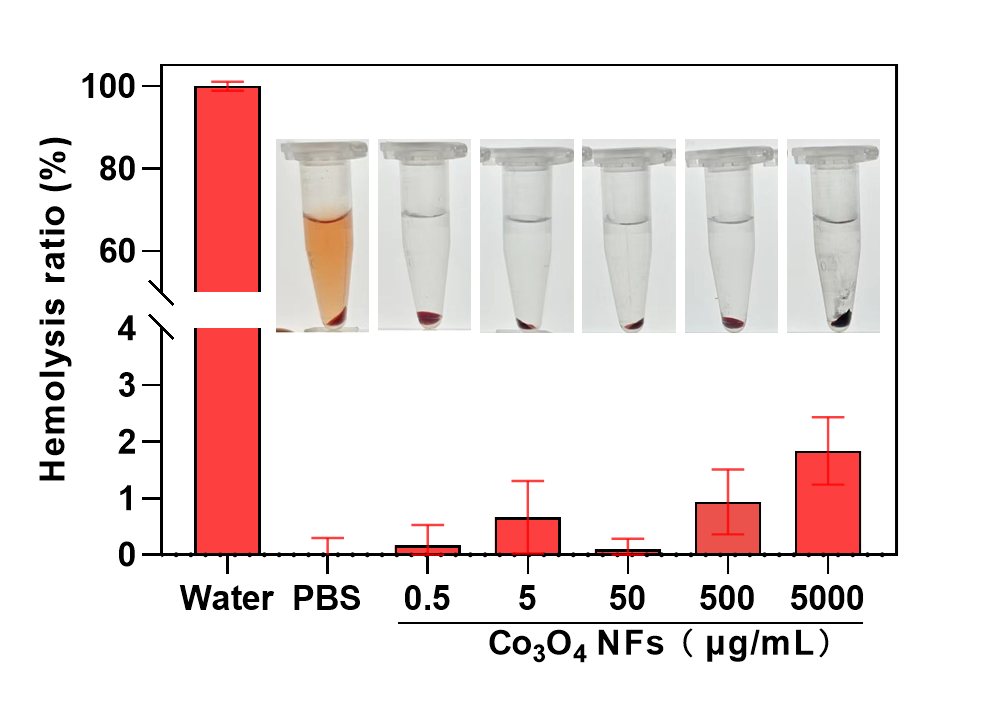


**Figure S6. Hemolysis ratios of RBCs treated with different concentrations of Co_3_O_4_ NFs.**


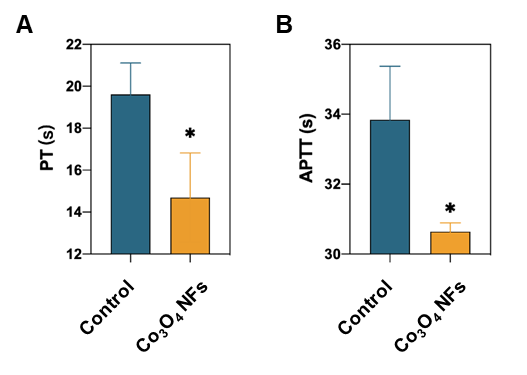


**Figure S7. Clotting time PT (A) and APTT (B).**


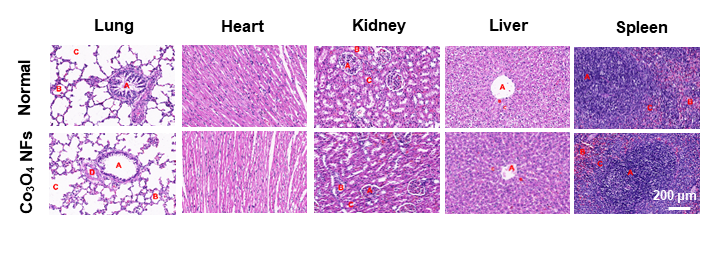


**Figure S8. H&E organ toxicity analysis of SD rats after 14 days of treatment with 50 μg/mL Co_3_O_4_ NFs**. Lung: respiratory bronchioles (A), interlacing alveoli (B), alveolar sacs formed by multiple open alveoli (C) and blood vessels (D); kidney: renal tubules and glomeruli (A), distal convoluted tubules (B), proximal convoluted tubules (C); liver: central vein (A); spleen: white pulp (A), red pulp (B), marginal zone (C).
